# Removal of penicillin G from aqueous phase by Fe^+3^-TiO_2_/UV-A process

**DOI:** 10.1186/2052-336X-12-56

**Published:** 2014-03-05

**Authors:** Mansooreh Dehghani, Simin Nasseri, Mohammad Ahmadi, Mohammad Reza Samaei, Amir Anushiravani

**Affiliations:** 1 Department of Environmental Health Engineering, School of Health, Shiraz University of Medical Sciences, Shiraz, Iran; 2 Department of Environmental Health Engineering, School of Public Health, and Center for Water Quality Research, Institute for Environmental Engineering, Tehran University of Medical Sciences, Tehran, Iran; 3 Department of Internal Medicine, Shiraz University of Medical Sciences, Student Research Center, Shiraz, Iran

**Keywords:** Antibiotic, Penicillin G, Fortified titanium dioxide with Fe^+^, Nano-photo catalyst removal

## Abstract

**Background:**

Anomalous use of antibiotics and their entrance into the environment have increased concerns around the world. These compounds enter the environment through an incomplete metabolism and a considerable amount of them cannot be removed using conventional wastewater treatment. Therefore, the main objectives of this research are evaluation of the feasibility of using ultraviolet radiation (UV-A) and fortified nanoparticles of titanium dioxide (TiO_2_) doped with Fe^+3^ to remove penicillin G (PENG) from aqueous phase and determining the optimum conditions for maximum removal efficiency.

**Results:**

The results showed that the maximum removal rate of penicillin G occurred in acidic pH (pH = 3) in the presence of 90 mg/L Fe^+3^-TiO_2_ catalyst. In addition, an increase in pH caused a decrease in penicillin G removal rate. As the initial concentration of penicillin G increased, the removal rate of antibiotic decreased. Moreover, due to the effect of UV on catalyst activation in Fe^+3^-TiO_2_/UV-A process, a significant increase was observed in the rate of antibiotic removal. All of the variables in the process had a statistically significant effect (p < 0.001).

**Conclusion:**

The findings demonstrated that the antibiotic removal rate increased by decreasing pH and increasing the amount of catalyst and contact time. In conclusion, Fe^+3^-TiO_2_/UV-A process is an appropriate method for reducing penicillin G in polluted water resources.

## Introduction

Antibiotic refers to a material that can be used for the elimination of microorganisms, such as bacteria, fungi, and parasites. Up to now, 250 antibiotics have been recorded for human, livestock, and plant consumption. The annual consumption rate of antibiotics has been estimated to be around 100000–200000 tons in the world [1]. Antibiotics are among the most beneficent drugs, however, they have potential harmful effects on environment, including entrance into soil and water resources and causing the development of antibiotic resistance microorganisms [2]. In addition, the residual antibiotics remain in the edible tissues of the animals [3].

Penicillin G is a common antibiotic which is used for treatment of different kinds of infectious diseases (Table 1). The antibiotic penicillin G is soluble in water and its mechanism of action is the destruction of bacteria’s cell wall by preventing peptidoglycan production [4].

**Table 1:** **Some physical and chemical properties of penicillin G**[4]

**Biological half-life**	**Solubility**	**Excretion method**	**Efficiency mechanism**	**Molecular weight**	**Chemical formula**
30-60 minutes	Soluble in water completely	Kidney	Prevention from wall -cell synthesis	372.48	C_16_H_17_KN_2_O_4_S

Antibiotics and their metabolites have been detected in surface water and ground water resources and drinking waters in the range of nanogram/L to microgram/L concentrations. However, these compounds cannot be effectively removed by conventional processes such as biological filtration, adsorption with activated carbon and reverse osmosis [5]. These methods can only transfer pollution from one phase to another [6]. On the other hand, advanced oxidation processes (AOPs), including UV/ZnO, UV/TiO_2_, and UV/H_2_O_2_[7], are efficient environmental friendly methods in which hydroxyl radicals (OH°) are used to oxidize recalcitrant organic pollutants and convert them to harmless end-products such as H_2_O and CO_2_[3].

Nanoparticles of metal oxides have a high rate of surface to volume ratio and can adsorb a large amount of materials [8]. Because of non-toxicity, low price, availability, chemical stability, and high light activity, titanium dioxide is used as the most usual semiconductor photo-catalyst for the removal of contaminants from water and air [9]. In spite of its many benefits, titanium dioxide has its own disadvantage that includes the relatively high speed in recombination of electrons and producing holes by the light activity (wavelengths <400 nm). Therefore, in order to increase its photocatalystic performance and also to prevent the recombination of electron–hole, the catalyst was doped with Fe^+3^[10,11]. Fe^+3^ metal ion has a half-full electron configuration [12] and can be replaced in TiO_2_ mesh because of having an ionic radius close to titanium. Moreover, it prevents the recombination of electrons and increases the activity of the catalyst by creating a surface trap for electrons and the holes formation as well [13].

Giraldo et al. used a photocatalystic system with TiO_2_ and showed that the antibiotics were changed into different compounds with lower toxicity and no antimicrobial properties [14]. Furthermore, Dimitrakopoulou et al. [15] demonstrated the effectiveness of TiO_2_/UV-A photocatalystic process for the removal of amoxicillin. They concluded that the removal rate of the antibiotic depended on the initial concentration of the amoxicillin and TiO_2_/UV-A catalyst [15]. Peterson’s study observed that nanoparticles of TiO_2_ removed significant amount of penicillin from the aqueous phase at acidic pH [4].

Many studies have been conducted on the removal of antibiotics using AOPs process. However, to date, no studies have used the application of UV light and fortified titanium catalyst doped with iron (Fe^+3^-TiO_2_/UV-A) for the removal of penicillin G. In recent years, misuse and arbitrary consumption of drugs, especially antibiotics, have become one of the basic challenges in health issues in Iran. Iran ranks first in antibiotic consumption worldwide, in which penicillin G has been widely used [16]. Moreover, there is a concern regarding contamination of water resources and its effect on people's health and the environment. Therefore, the objectives of the study were to (i) evaluate the feasibility of using fortified nanoparticles of titanium dioxide doped with Fe^+3^ (Fe^+3^-TiO_2_) using cell-gel method in conjunction with UV radiation in removing penicillin G from the aqueous phase and (ii) determine the optimum conditions for maximum removal efficiency.

## Materials and methods

The experiments were carried out in duplicates in the batch mode. The study parameters were pH, reaction time, catalyst dose, and initial antibiotic concentration. Factorial design was used for the analysis of the parameters and their interaction effects were studied as well. To reduce the scatter in the data, log of transformation and geometric mean were used.

### Chemicals and analytical method

Penicillin G with 99% purity was purchased from Sigma-Aldrich Company (USA). Other chemical products were purchased from Merck (Germany). UV lamp (F8T5) with the length of 25 Cm, 8 W and 356 nm wave length, (Hitachi, Japan) was used as the radiation source.

**Figure 1:**
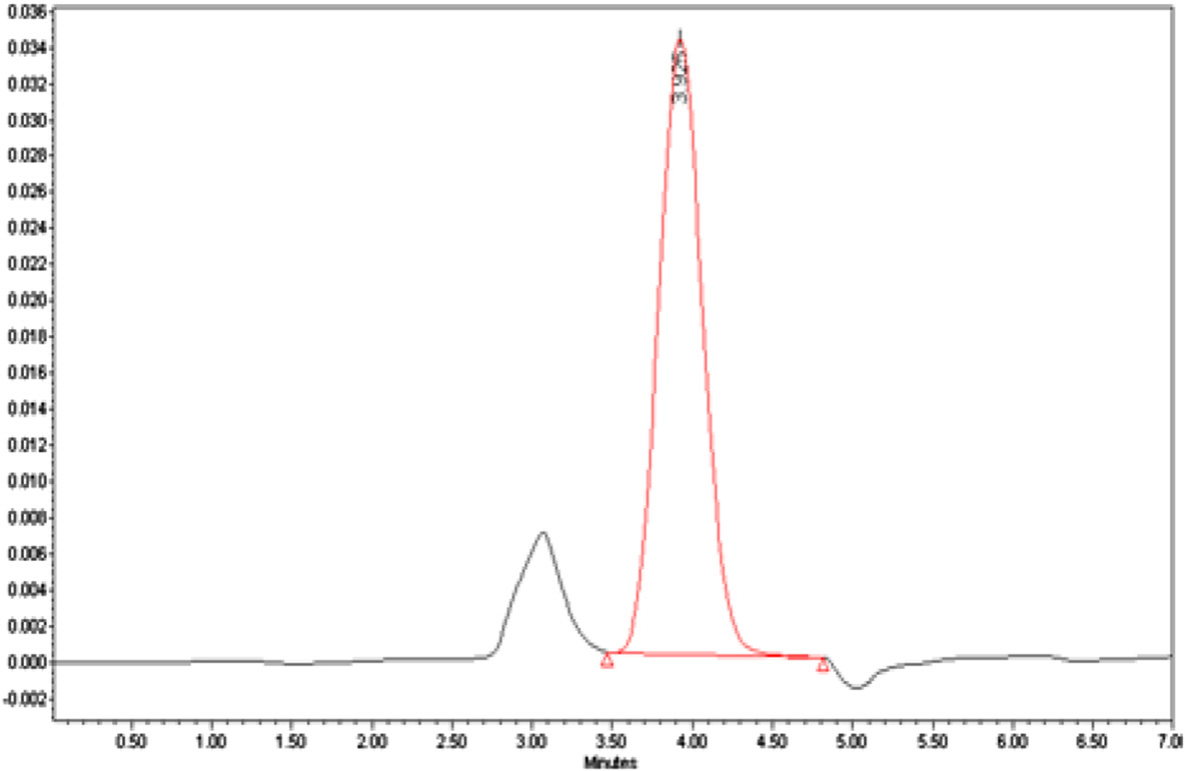
Penicillin G chromatogram.

For penicillin G detection in the aqueous phase a Waters Model high performance liquid chromatography (HPLC) (Waters YL9100HPLC SYSTEM, USA) system with C_18_ columns (CP-SIL 5 CB column model, 250*4.6 mm, 5 μm) was calibrated and tested prior to injection of the samples. The mobile phase included methanol and water (20/80 V/V) with a flow rate of 0.5 mL/min. A UV absorbance detector at 210 nanometer wave length was used to detect penicillin G in the samples. The retention time for the antibiotic was 7 minutes. The detection limit for the sample was 1 nanogram/L. Penicillin G chromatogram is presented in Figure 1.

Scanning Electroscope Microscope (SEM) (EM3200, KYKY Company, China) was used to determine the morphology and the mean diameter of the catalyst’s particles.

### Fortified catalyst preparation

Cell-gel method was used to prepare Fe^+3^-TiO_2_ nanocatalyst powder. A first, ferrous nitrate was dissolved in half of propanol (121.775 mL) and completely mixed using a homogenizer. After 15 minutes, another half of propanol (121.775 mL) was mixed with titanium tetraisopropoxide (TTIP) (62.77 ml) and then the mixture was added very slowly (in 75 minutes) to the former solution to prepare the sol. Meanwhile, deionized distilled water (8.33 mL) was added to the solution as well. Thirty minutes after the addition of propanol to TTIP, the pH was adjusted to 3 by nitric acid. All the processes were performed in mixing mode using the homogenizer. Then, the resulting solution was placed on the magnetic mixer for 24 hours to form jelly. After that, the formed jelly was put in the oven at 80°C for 10 hours to evaporate alcohol. To activate the catalyst, the jelly was put in oven at 500 ± 50°C for 2 hours. The activated catalyst was put in desiccators until it was cool. Finally, the catalyst was powdered [17].

### Reactor specification

**Figure 2:**
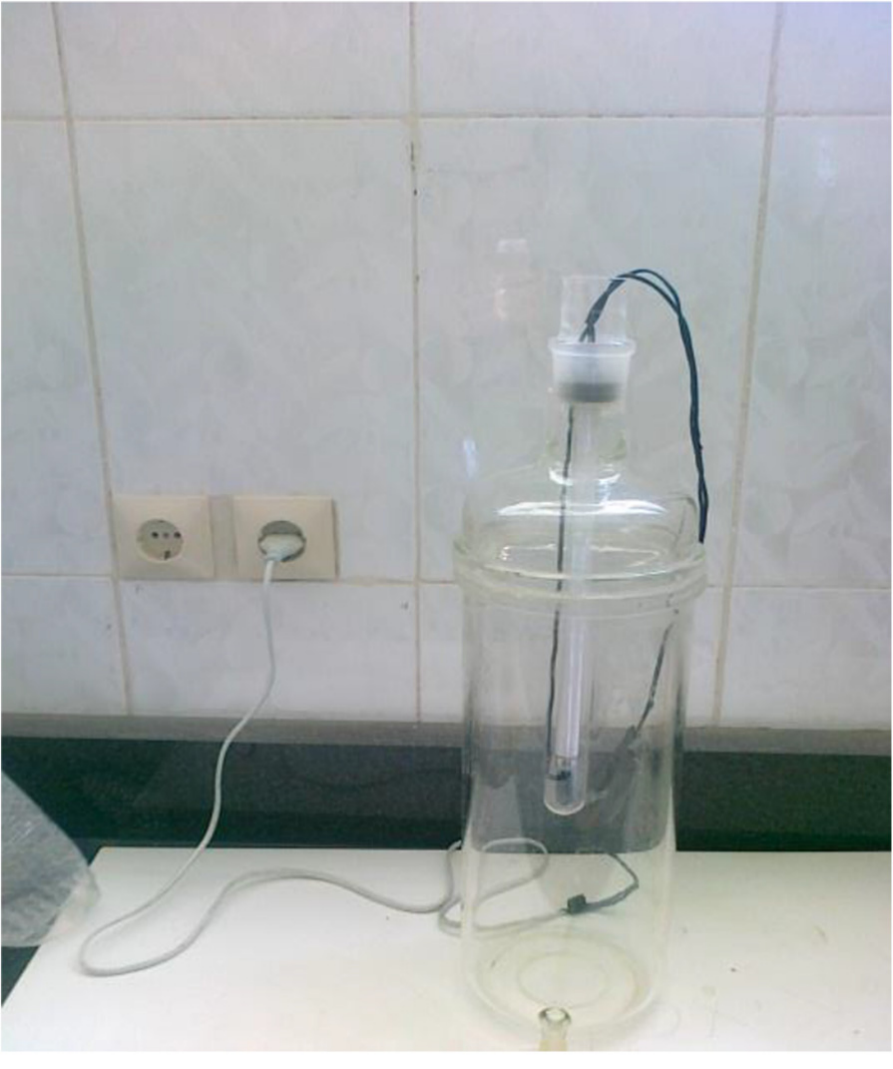
Photochemical reactor.

The specification of photochemical reactor is shown in Figure 2. The experiment was performed in a 2-liter volume reactor. Test was performed in a closed glass reactor with adjustable mixer. The source of radiation was a UV lamp which was protected by a Quartz tube with the height of 30 cm and inner diameter of 5 cm. The UV radiation source was immersed in the solution for better radiation. The whole system was wrapped in an aluminum foil in order to prevent reflection.

### Effects of pH and contact time on the removal rate of penicillin G by Fe^+3^-TiO_2_/UV-A process

To measure the influence of different parameters on the removal rate of penicillin G by Fe^+3^-TiO_2_/UV-A process in the aqueous phase, different pH from 3–11 (interval of 4) with two replications was used at the antibiotic concentration (10, 25, and 45 mg/L), catalyst concentrations (30, 60, and 90 mg/L), and the contact time of 30, 60, 90, and 120 minute intervals. A blank without catalyst Fe^+3^-TiO_2_ was also used for all the experiments. At the end of each run, EBA20 centrifuge (Hettich Company, Germany) was used at 6000 rpm for 15 minutes to separate the catalyst particles from penicillin G solution. Then, the samples were passed through a Whatman filter cellulose acetate membrane with 0.45 micron pore size (Germany). After that, the residual of penicillin G was measured using HPLC. All the experiments were done in two replications in the presence of the control samples.

## Results and discussion

**Figure 3:**
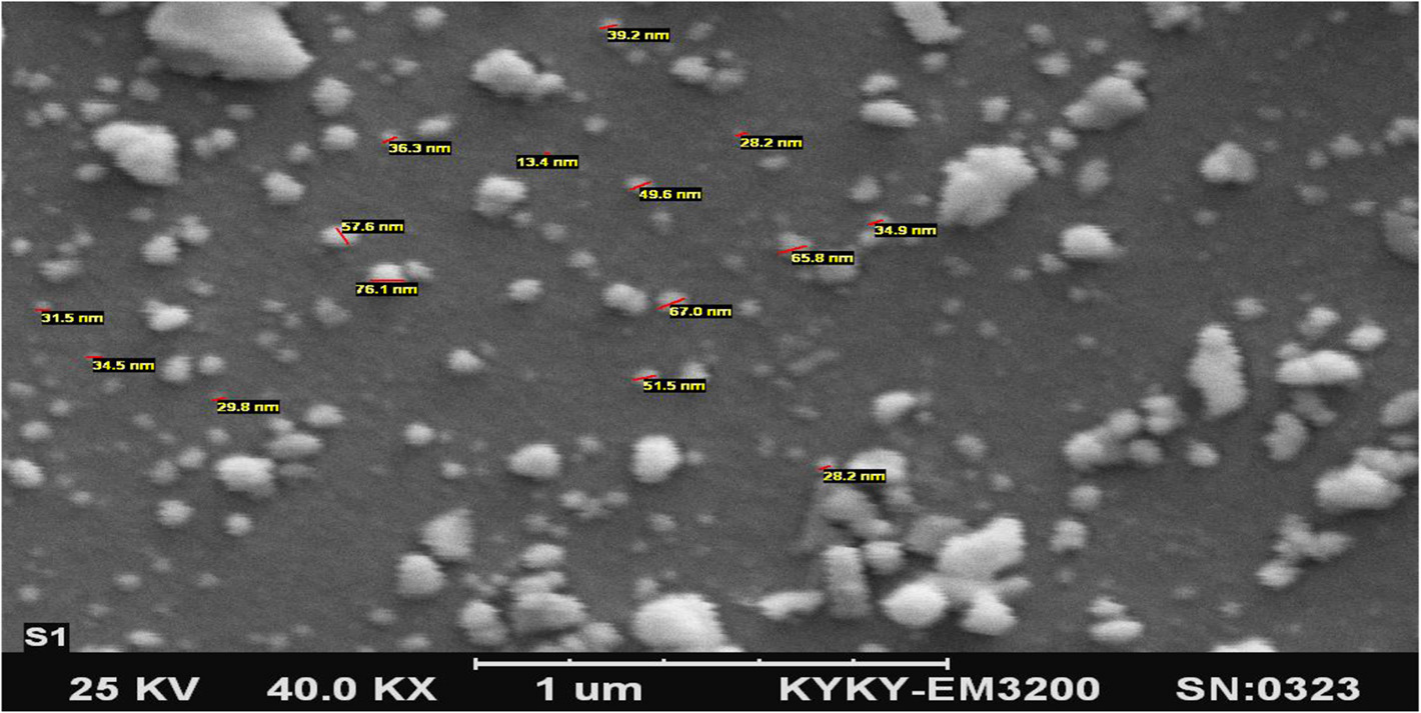
**Image of catalyst Fe**^**+3**^**-TiO**_**2 **_**using Scanning Electroscope Microscope (SEM).**

The structure of the catalyst’s crystal was determined using D8 Advanced Ray Diffractometer (XRD) (Bruker AXS, Germany). The mean diameter of the catalyst’s particles was less than 50 nm using SEM (Figure 3).

### Effects of pH and contact time on the photocatalystic removal rate of penicillin G by Fe^+3^-TiO_2_/UV-A process

**Figure 4:**
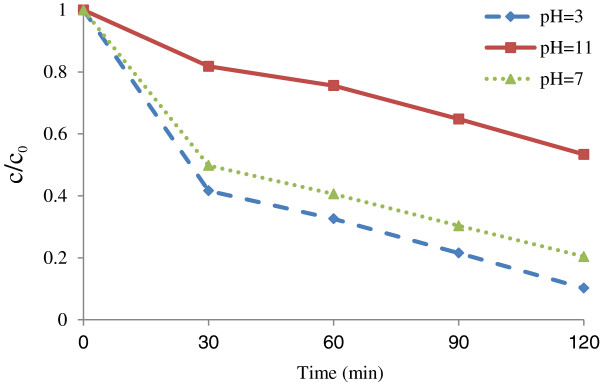
**Effect of pH on photocatalystic removal of penicillin G by Fe**^**+3**^**-TiO**_**2**_**/UV-A process (Fe**^**+3**^**- TiO**_**2**_ **= 60 mg/L, penicillin G =25 mg/L).**

The variations of pH on the rate of adsorption of penicillin G by Fe^+3^-TiO_2_/UV-A process are shown in Figure 4. Data regarding the effect of pH showed that as pH increased from 3.0 to 11, the rate of penicillin G reduction decreased (Figure 4). We showed that a pH of 3 was optimal for penicillin G degradation. The reduction rate was more than 71% in this case. On the other hand, the minimum removal rate of the antibiotic in the aqueous phase was related to pH = 11 (28.48%). According to regression analysis it can be concluded that there was a significant difference between pH and penicillin G removal rate (p < 0.001).

pH is one of the most important factors affecting the efficiency of chemical and biological processes especially in advanced oxidation process. Advanced oxidation processes are developing technologies for removing pollutants from atmosphere, water, and wastewater [18]. pH has a considerable effect on the solubility of antibiotics, catalyst surface charge, as well as the mechanism of hydroxyl radical production [19]. The feasibility of hydroxyl radical production and oxidation efficiency also depend on pH. As pH increased, the removal rate of penicillin G decreased due to the reduction in hydroxyl radical oxidation potential [20]. Furthermore, high concentrations of H^+^ ions in acidic environments lead to the formation of H^0^ radicals and, by using the available oxygen in solution, form the HO_2_° radicals that are eventually converted to OH° radicals. AOPs are based on the formation of hydroxyl radicals potential that oxidize the pollutants [18,21]. Additionally, high pH values intensify the formation of HO_2_^-2^ ions and destruction of hydroxyl radicals by carbonate and bicarbonate ions. The reduction rate of penicillin G reduced at higher pH, because of the formation of insoluble compounds which in turn reduced the intensity of UV radiation and the potential of hydroxyl radical production as well. Other studies also demonstrated that better removal of antibiotic occurred at lower pH [1,3,6].As shown in Figure 4, the removal rate of penicillin G by nano photocatalystic process increased as the contact time increased (30–120 min). After 90 min equilibration time, its rate became almost constant (90–120 min). Regression analysis showed that there was a significant difference between contact time and the antibiotic removal rate (p < 0.001).

**Figure 5:**
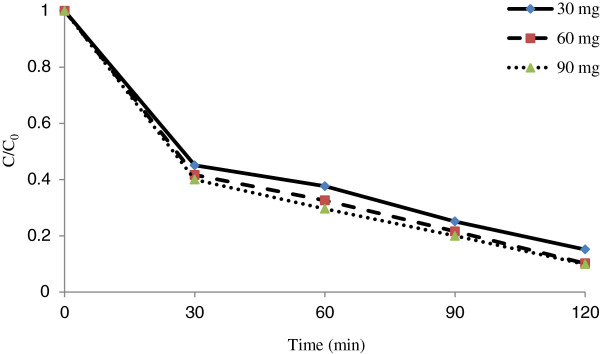
**Effect of Fe**^**+3**^**-TiO**_**2 **_**dose on photocatalystic removal of penicillin G by Fe**^**+3**^**-TiO**_**2**_**/UV-A process (pH = 3 and penicillin G = 25 mg/L).**

**Figure 6:**
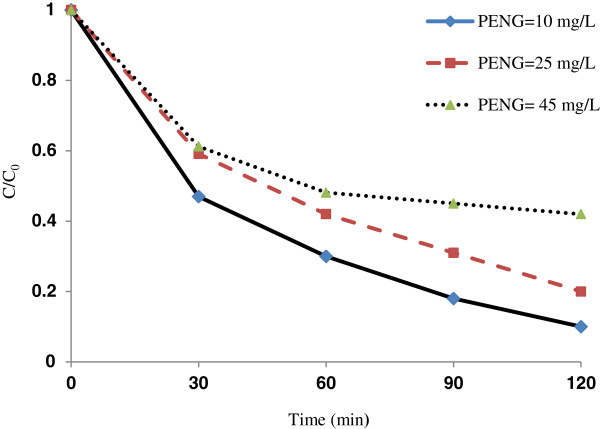
**Effect of initial antibiotic concentration (PENG) on photocatalystic removal of penicillin G by Fe**^**+3**^**-TiO**_**2**_**/UV-A process (pH = 3 and Fe**^**+3**^**-TiO**_**2**_ **= 60 mg/L).**

Determining the equilibration time is another important factor to achieve the maximum rate of antibiotic reduction in the aqueous phase [2]. According to the results illustrated in the current study, at first the photocatalystic rate of penicillin G reduction increases very fast as the contact time increased. After that, its rate becomes slower until it reached a plateau (Figures 4, 5 and 6). This phenomenon may be related to the presence of many vacant sites on the catalyst surface to form hydroxyl radicals. After that, the remaining sites were not easily accessible to form OH°. At equilibrium, the degradation reached a plateau. If the reaction time exceeds equilibrium, the process will be no longer cost-effective and economical [22]. Basically, an optimal contact time is a very important parameter for any chemical reaction. Based on our study, 120 min reaction time is optimal for penicillin G degradation (Figure 6). Dimitrakopoulou et al. also found that the removal of amoxicillin using AOPs had an initial steep slope reaching a plateau with a relative slow equilibration at 90 minutes [15].

### Effects of Fe^+3^-TiO_2_ dose and contact time on the photocatalystic removal rate of penicillin G by Fe^+3^-TiO_2_/UV-A process

Penicillin G photocatalystic removal rate increased as the applied catalyst (Fe^+3^-TiO_2_) dose increased from 30 to 90 mg/L. According to Figure 5, the optimal of Fe^+3^-TiO_2_ catalyst dose and the reduction rate of penicillin G are 90 mg/L and 90.5%, respectively. According to Figure 5, the rate of the antibiotic removal for different catalyst dose was in the range of 74.9 to 90.5%. According to regression analysis it can be concluded that there was a significant difference between catalyst dose and penicillin G removal rate (p < 0.001).

In our study (Fe^+3^-TiO_2_/UV-A), increasing Fe^+3^-TiO_2_ catalyst dose increased the production rate of hydroxyl radical. By increasing the catalyst the metal active surface increased as well. The reduction of the pollutant is basically proportional to the formation of hydroxyl radicals on the surface of catalyst. An increase in the amount of catalyst increased the number of the absorbed photons which in turn increased the activated sites on the catalyst surface [17]. Therefore, the amount of the adsorbed penicillin G would also increased. Penicillin G removal was associated with the available concentration of the catalyst in the solution and antibiotic removal increased linearly with an increase in the catalyst concentration. Similar results were also obtained for the removal of penicillin using titanium dioxide nanoparticles [4].

### Effects of initial antibiotic concentration of penicillin G (PENG) and contact time on the photocatalystic removal rate of penicillin G by Fe^+3^-TiO_2_/UV-A process

The effect of initial antibiotic concentration of penicillin G on the photocatalystic removal rate at the optimal condition (pH = 3, Fe^+3^-TiO_2_ = 60 mg/L) is shown in Figure 6. Antibiotic removal rate decreased from 95 to 51% as the initial penicillin G concentration increased from 10 to 45 mg/L. Regression analysis showed that there was a significant difference between initial antibiotic concentration on penicillin G and the antibiotic removal rate (p < 0.001).

The antibiotic’s initial concentration plays a major role in many photocatalystic processes. We demonstrated that as the initial penicillin G concentration increased the photocatalystic removal rate of antibiotic decreased (Figure 6). Since the concentration of the radicals produced was the same in all samples in the photocatalystic process, the feasibility of removing antibiotic was lower. Higher availability of hydroxyl radicals may result in higher rate of penicillin G oxidation. Therefore, samples with lower initial penicillin G concentration with the same amount of hydroxyl radicals have a higher chance of removal. Our results agree with Fang et al. on metronidazole removal [23].

### Effects of UV radiation on the photocatalystic removal rate of penicillin G by Fe^+3^-TiO_2_/UV-A process

**Figure 7:**
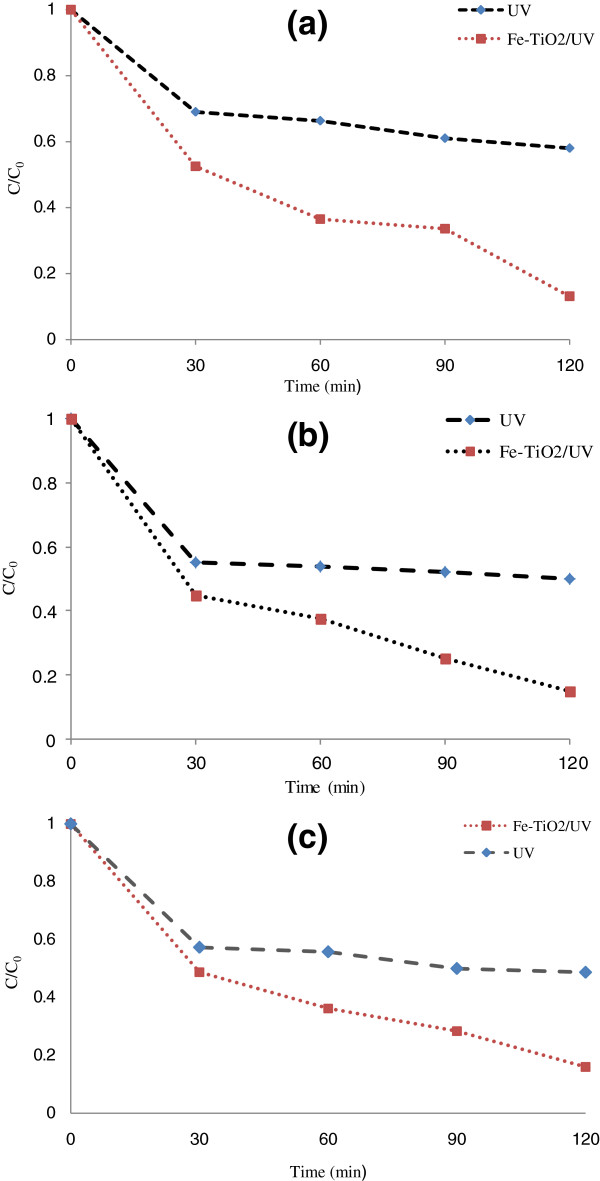
**Effects of UV radiation on the photocatalystic removal rate of penicillin G by Fe**^**+3**^**-TiO**_**2**_**/UV-A process at different conditions (a) pH = 3, penicillin G concentration (PENG) =10 mg/L, (b) pH = 3, penicillin G concentration (PENG) =25 mg/L, (c) pH = 3, penicillin G concentration (PENG) =45 mg/L.**

In order to assess the effect of UV radiation on Fe^+3^-TiO_2_/UV-A photocatalystic removal, the experiments were performed in optimal pH (pH = 3) at different concentrations of penicillin G (10 mg/L, 25 mg/L, and 45 mg/L) without the use of catalyst Fe^+3^-TiO_2_. Figure 7(a-c) demonstrated that at low concentrations of penicillin G in the absence of any catalyst, using UV radiation greatly affected the removal rate of antibiotic and decreased its reduction (38%). At high concentration of penicillin G, however, the use of Fe^+3^-TiO_2_ nanocatalyst led to a significant increase in the removal rate (84%). Regression analysis showed that there was a significant difference between UV radiation and the antibiotic removal rate (p < 0.001).

## Conclusion

In conclusion, the results of this research showed that Fe^+3^-TiO_2_/UV-A process had significantly reduced penicillin G in liquid phase. The rate of removal showed an initial increase, reaching a plateau with a relative slow rate. Removal of the antibiotic increased with a decreasing initial concentration of penicillin G, increasing with the catalyst dose. Moreover, penicillin G removal in the aqueous solution was relatively high at pH = 3 and contact time = 120 min. According to the current study, the reduction rate of the penicillin G from aqueous solutions was more than 90% in optimal conditions. Therefore, Fe^+3^-TiO_2_/UV-A process is as an efficient and cost-effective method to remove penicillin G from water resources and make it feasible to reduce antibiotic concentration in drinking water to the desirable level.

## Competing interests

The authors declare that they have no competing interests.

## Authors’ contributions

The overall implementation of this study including design, experiments and data analysis, and manuscript preparation were the results of the corresponding author’s efforts. All authors have made extensive contribution into the review and finalization of this manuscript. All authors read and approved the final manuscript.
